# Non-destructive quality assessment and species identification of blood tofu using portable visible/near-infrared spectroscopy

**DOI:** 10.3389/fnut.2026.1877866

**Published:** 2026-07-03

**Authors:** Ming Li, Wenqiang Guan, Pingyan Xin, Xiaoxiao Li, Dingqiang Lu

**Affiliations:** Tianjin Key Laboratory of Food Biotechnology, School of Biotechnology and Food Science, Tianjin University of Commerce, Tianjin, China

**Keywords:** blood tofu, competitive adaptive reweighted sampling, partial least squares discriminant analysis, partial least squares-linear discriminant analysis, quality assessment, species identification, visible/near infrared spectroscopy

## Abstract

**Introduction:**

The identification and quality monitoring of blood-based meat by-products represent critical challenges in the meat industry. This study investigates the feasibility of portable visible/near-infrared (Vis-NIR) spectroscopy as a rapid, non-destructive analytical tool for quality assessment and species identification of duck and pig blood tofu during refrigerated storage at 4°C. The development of reliable spectroscopic methods is essential for ensuring product integrity and preventing fraud in meat processing.

**Methods:**

Blood tofu samples from duck and pig sources were stored at 4°C for 15 days. A total of 180 blood tofu samples, comprising 90 duck blood tofu and 90 pig blood tofu samples, were utilized for quality assessment and species identification. Physicochemical parameters including total volatile basic nitrogen (TVB-N) and pH, color coordinates (CIE L^*^a^*^b^*^ system), and textural properties (hardness) were monitored throughout the storage period to establish reference quality data. Partial least squares (PLS) regression models were developed for quantitative prediction of quality parameters. For classification purposes, partial least squares-linear discriminant analysis (PLS-LDA) and partial least squares discriminant analysis (PLS-DA) models were constructed, with competitive adaptive reweighted sampling (CARS) employed for optimal wavelength selection.

**Results and Discussion:**

PLS regression models demonstrated satisfactory predictive performance for color parameters, achieving correlation coefficients approaching or exceeding 0.90 for L^*^ and a^*^ color coordinates. Based on intrinsic spectral fingerprints, the CARS-optimized model achieved highly accurate discrimination between blood tofu derived from duck and pig, with an area under the curve (AUC) of 1.00, demonstrating exceptional capability for species identification. Most significantly, the PLS-LDA and PLS-DA classification models successfully discriminated between edible and inedible blood tofu samples with high accuracy. Compared with CARS-PLS-DA, the CARS-PLS-LDA model possesses a more parsimonious structure and clearer class discrimination. Portable Vis-NIR spectroscopy proves to be a powerful analytical tool for comprehensive quality monitoring of blood-based meat by-products. The technique enables rapid assessment of freshness indicators, feasibility of shelf-life assessment, and capability in species identification in the meat by-products industry under refrigerated storage.

## 1 Introduction

Livestock and poultry blood is one of the main by-products after animal slaughter. At the same time, the blood is rich in nutrients. The protein content is generally close to or higher than that of muscle tissue of the same species of livestock and poultry, and the amino acid composition of the protein is more balanced. Blood also contains a variety of minerals. Potassium, sodium, magnesium, and phosphorus mostly exist in the form of inorganic salts. Iron is found in the form of porphyrin iron, ferritin and other organic iron (1). These characteristics can make the nutrients in the blood directly absorbed by the body, and the utilization rate is high. Moreover, due to homology, protein components in the blood of livestock and poultry do not cause food allergy in humans (2). Therefore, using livestock and poultry blood as food raw material can not only reduce environmental pollution, but also meet the nutritional model recommended by FAO/WHO experts (3).

Blood-based food is made from the blood of livestock and poultry, supplemented with appropriate amount of water and appropriate food additives and seasonings. Blood-based dishes are deeply embedded in many traditional cuisines, such as blood sausages, blood tofu, soups and stews. Blood tofu is made via simple heating of the fresh edible animal blood and widely distributed in the supermarkets or restaurants in China (4). However, visual inspection is insufficient for accurate differentiation due to the high similarity in the appearance of blood tofu products from different animal species. This poses a significant risk of economically motivated adulteration, where high-value products, such as duck blood tofu, are substituted with lower-cost alternatives like pig blood tofu (5). In addition, the special flavor of this kind of food and the consumption after heating and processing lead to the inability to identify poor quality blood tofu. This situation will eventually lead to food insecurity. The traditional quality testing methods of blood tofu mainly include sensory evaluation, physical and chemical analysis and microbiological detection. At present, the detection methods of sensory indexes of blood tofu are mainly based on human operation, which itself is uncertain. At the same time, the detection methods of physical and chemical indicators have some disadvantages, such as high reagent consumption, complex operation, time-consuming and laborious. In the actual situation, this traditional detection method is not very practical. Therefore, it is urgent to develop new methods for the detection of quality parameters and species identification of edible animal blood products to regulate the market of animal blood products, prevent food fraud, and protect the interests of consumers.

In recent years, portable visible/near infrared (Vis-NIR) spectrometer, as a simple, fast and effective analysis instrument, is being applied in many fields in the way of industrial chain, such as agriculture, petrochemical, pharmaceutical and food industry (6–11). The visible spectrum mainly reflects the characteristics of some chromophores and co-chromophores in the material molecules (12). The near-infrared spectrum mainly carries the information of frequency doubling and frequency joining of molecular hydrogen-containing groups, such as O-H, N-H, C-H and other hydrogen-containing groups (13). Since this information can be loaded onto the near-infrared spectrum, this spectral region is capable of analyzing samples with a wide range of chemical structures. Meanwhile, the NIR spectroscopy detection technology can achieve pollution-free, non-reagent consumption, and non-contact rapid detection of samples. Because the Vis-NIR spectroscopy method combines the advantages of both wavelength bands, with low equipment cost and simple operation methods, this analytical detection method has gradually become familiar to the industry community. In recent years, a variety of general and special instruments have been developed (14, 15).

Currently, research on the application of spectroscopy to edible blood-based by-products has primarily focused on species adulteration, pesticide residue detection, and the analysis of processing characteristics. Han et al. (16) developed a practical technique for the authentication of the duck blood tofu binary and ternary adulterated with cow and pig blood-based gel using Fourier transform near infrared spectroscopy (FT-NIR). Huang et al. (17) established a rapid detection method for sulfamethazine residues by combining surface-enhanced Raman spectroscopy technology. E. Saguer (18, 19) employed FT-NIR to analyze the denaturation and gelation phenomena of porcine blood plasma during processing. However, systematic and comprehensive studies on the quality parameters testing and identification of species and edibility of blood tofu during refrigerated storage using portable Vis-NIR spectroscopy are still relatively scarce. There is still some room for improvement in the spectral data processing methods, the versatility and accuracy of the model, which urgently needs to be further studied.

In this thesis, a portable Vis-NIR spectrometer was used to develop a rapid and non-invasive method for the determination of the physicochemical, color, and textural properties of edible blood tofu, including total volatile base nitrogen (TVB-N), pH, hardness and color. This study also investigated the feasibility of portable Vis-NIR for identification of species and edibility. Combined with spectral technology, attempts were made to explore the linkage mechanism between the multi-index and spectral representation. Then it provided a theoretical basis for the establishment of model.

## 2 Materials and methods

### 2.1 Preparation of edible blood tofu samples

In this study, fresh blood from ducks and pigs was used to prepare edible blood tofu respectively. Duck and pig fresh blood samples were provided by the local slaughterhouse in Tianjin, China. The 10 mL of 1% sodium citrate solution was added to every 50 mL of fresh blood and stirred thoroughly for 30 s. And then, the blood mixtures were transported to the laboratory in an ice-filled box. Each 15 mL of the blood mixture was mixed with 1 mL of 1% calcium chloride solution and stirred for 30 s at room temperature. The stirred liquid was set for 10 min and then subjected to water bath heating for 30 min. The solidified solid was the edible blood tofu sample used in this experiment (20). The prepared blood tofu samples were cut into uniform cubes of 6.0 cm × 6.0 cm × 3.0 cm (length × width × height), stored at 4°C, and the storage time was recorded. This specific size was chosen to ensure consistent spectral acquisition and to minimize the edge effect. The samples were packed in sealed plastic containers to prevent moisture loss and cross-contamination during storage. The storage duration (15 days) was selected to cover the typical shelf-life of fresh blood products, allowing for the assessment of quality deterioration.

### 2.2 Vis-NIR spectroscopy measurements

In this study, fresh blood from ducks and pigs was considered independently. The quality prediction models of edible blood tofu of two species were established. For each sampling day, 15 independent samples of each species were collected for analysis (15 duck blood tofu samples and 15 pig blood tofu samples). Samples were collected at 3-day intervals. Consequently, a total of 90 samples were obtained for duck blood tofu and 90 for pig blood tofu throughout the 15-day experiment.

**Figure 1:**
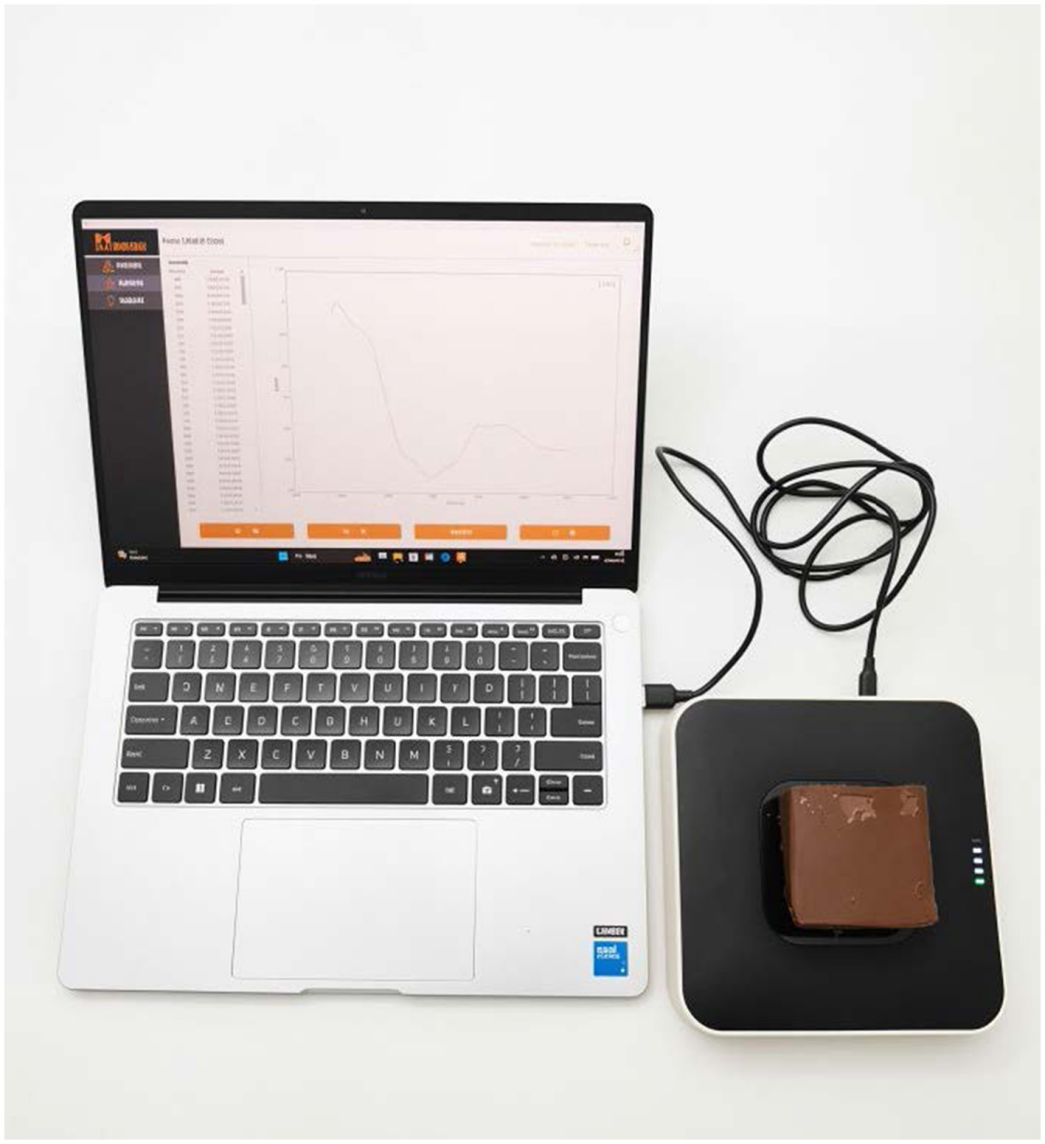
Vis-NIR spectroscopy measurement of blood tofu.

Figure 1 illustrates the Vis-NIR spectral acquisition method of blood tofu sample. The spectrum collection was carried out using a portable visible/near-infrared spectrometer (N110, Hanon, Shandong, China) equipped with a spherical grating monochromator with micro-electromechanical technology. Before the collection of the sample's spectrum, the air was measured as reference. Then, the separated samples were placed in the circular probe for spectral measurement. The integration time for the sample was 50 ms and the reference integration time was fixed at 10 ms. For each spectrum, an average of 10 scans was performed at a resolution of 2 nm over the 592–1102 nm wavelength range, which includes the visible and NIR regions. The absorbance spectra were converted into the $Log\left(\frac{1}{R}\right)\;$using the following Equation 1:


$$\begin{array}{l} Log\left(\frac{1}{R}\right)\;=\;Log\left[\left(I_{R}-I_{D}\right)/\left(I_{S}-I_{D}\right)\right] \end{array}$$ (1)

The $Log\left(\frac{1}{R}\right)\;$was calculated automatically by taking the raw sample spectral energy (*I*_*S*_), the dark spectral energy (*I*_*D*_) and the reference spectral energy (*I*_*R*_). The spectra and reference values were measured at exactly the same locations. For each sample, spectra were collected in triplicate from the same detection location, and the average spectrum was calculated to represent the sample.

After acquiring the spectra, the same area of the sample's reference was analyzed instrumentally and chemically.

### 2.3 Reference determinations

#### 2.3.1 Color

The color attributes of edible blood tofu samples were quantitatively determined using a CR-400 chromameter (Konica Minolta Sensing, Inc., Tokyo, Japan). Prior to colourimetric analysis, the instrument was standardized with a white calibration tile under ambient laboratory conditions (*Y* = 93.5, *x* = 0.3114, *y* = 0.3190). Color parameters were expressed in the CIE L^*^a^*^b^*^ color space as defined by the Commission Internationale de l'Éclairage (21), where L^*^ represents lightness, a^*^ denotes redness/greenness, and b^*^ indicates yellowness/blueness. For each sample, color measurements were performed at the identical location corresponding to the spectroscopic analysis region to ensure measurement consistency. All colourimetric determinations were conducted in triplicate, and the mean values with standard deviations were reported for statistical analysis.

#### 2.3.2 Hardness

The determination of sample hardness was performed referring to the method of Fan et al. (22) with slight modifications. The parameters of the texture analyzer were set as follows: a P36R probe, 50% compression, a pre-test speed of 2.0 mm/s, a test speed of 1.0 mm/s, and a post-test speed of 5.0 mm/s. The time interval between two compressions was 3 s, and the trigger force was set to Auto-5g. Each sample was measured in triplicate, and the mean value was calculated.

#### 2.3.3 pH value

The pH value of sample was determined directly using a pH meter (716 DMS Titrino, Metrohm, Herisau, Switzerland). The pH meter was calibrated using pH buffers (pH 4.01 and 7.00) before sample determination. The probe was inserted 2 cm into sample tissue, and then the data were recorded after the readings were stabilized.

#### 2.3.4 TVB-N value

In this experiment, the TVB-N value of the samples was measured by automatic Kjeldahl nitrogen determination instrument method according to the National Food Safety Standard of China (23). Ten grams of the crushed sample were placed into the distillation tube, and 75 mL of deionized water were added to it. This allowed the sample and the deionized water to be thoroughly mixed and dispersed. The mixture was left to soak for 30 min before being tested. Then, 1 g of magnesium oxide powder was added to the distillation tube and shaken. Next, it was placed in a semi-automatic Kjeldahl nitrogen analyser (8,200, FOSS). 30 mL of boric acid (20 g/L) was added to the receiving bottle, and the distillation time was set to 180 s. At the end of the reaction, 10 drops of mixed indicator containing 1 part of 1 g/L methyl red ethanol solution and 5 parts of 1 g/L ethanol solution were added to the receiving solution. Then, 0.1 mol/L hydrochloric acid standard titration solution was added dropwise until the receiving liquid turned purple (24). The TVB-N content of the mutton sample was calculated by the following Equation 2.


$$\begin{array}{l} X_{s}=\frac{\left(V_{1}-V_{2}\right)\times c\times 14}{m}\times 100 \end{array}$$ (2)

where *X*_*s*_ is the TVB-N content of the blood tofu sample. *V*_1_ and *V*_2_are the volumes of the sulfuric acid standard titration solution consumed by the experimental group and the blank control, respectively. *c* is the concentration of hydrochloric acid standard titration solution (mol/L), and *m* is the weight of the minced sample (g). The values of TVB-N were expressed as mg/100 g sample.

#### 2.3.5 Data processing

A total of 180 blood tofu samples, comprising 90 duck blood tofu and 90 pig blood tofu samples, were utilized. Before establishing the model, samples were divided into a calibration set and a prediction set in a ratio of 3–1 using the Kennard- Stone (KS) method (25). In order to enhance the prediction ability of the models and the qualitative interpretation of the spectra, some spectral pre-treatments were carried out, such as first derivative (1^st^), second derivative (2^nd^), multiplicative scatter correction (MSC), standard normal variate transformation (SNV) (26) and so on.

#### 2.3.6 Model development

In this study, the physicochemical and sensory properties of blood tofu from two species were considered independently. The prediction models for each index were developed using partial least squares (PLS) regression (27). In PLS algorithms, the leave-one-out cross-prediction technique is generally used to select optimal latent variables (LVs), being determined by the lowest root mean square error of the cross prediction value (RMSECV). The calibration and predictive ability of the model were assessed using the correlation coefficient (*R*), the root mean square error of calibration (RMSEC), the root mean square error of prediction (RMSEP), the ratio of prediction to deviation (RPD) and range error ratio (RER) (28). The result of the calibration was evaluated by RMSEC, RMSEP and *R*. A good model should have a lower RMSEC, RMSEP and higher *R*, RPD and RER, but also show a small difference between RMSEC and RMSEP (29). The PLS was established using Matlab2014a (Math works, Natick, MA, USA).

Meanwhile, this study used partial least squares–linear discriminant analysis (PLS-LDA) (30) method to distinguish species identification and edibility (suitability for consumption due to quality deterioration). The PLS-LDA method has proven to be effective in discriminant studies. This method reduces the number of dimensions using PLS and applies a LDA to the PLS components. The PLS algorithm was run on a sample spectrum matrix and a Y matrix to determine the PLS components, based on training observations only. The duck blood tofu and edible blood tofu samples were assigned a value of 1 for the Y matrix. The pig blood tofu and inedible blood tofu samples were assigned a value of −1 for the Y matrix. The PLS components were then computed for the test data set. Applying a classical LDA to the new components resulted in successful classification. Models were established using LVs, which were detected by cross validation (CV). The optimal number of LVs was estimated using 10-fold CVs for the models. The LVs were considered as new eigenvectors of the original spectra to reduce the dimensionality and compress the original spectral data. Concurrently, this study also employed the classical Partial Least Squares Discriminant Analysis (PLS-DA) to establish a baseline classification model (31). A comparative evaluation was conducted to investigate whether the PLS-LDA exhibited superior discriminative performance in distinguishing the physicochemical attributes of these two substances, as hypothesized.

The performance of the discriminant models was assessed based on sensitivity, specificity, classification accuracy and area under the curve (AUC) (32). For every possible classifier selected to discriminate samples, the four outcomes would be as follows: positive samples correctly classified as positive *T*_*P*_ (true positive,), positive samples erroneously classified as negative *F*_*N*_ (false negative), negative samples correctly classified as negative *T*_*N*_ (true negative) and negative samples erroneously classified as positive *F*_*P*_ (false positive). Sensitivity was defined as a true positive fraction, and specificity was defined as a true negative fraction. *C*_*a*_ (classification accuracy) was defined by the Equation 3:


$$\begin{array}{l} C_{a}\;=\;\frac{T_{P}+T_{N}}{T_{P}+T_{N}+F_{P}+F_{N}} \end{array}$$ (3)

To analyze the actual discriminative power of the classifier, a receiver-operating characteristic (ROC) curve is used to view the performance of the 2-class classifier. The ROC curve is a plot of “sensitivity” and “1-specificity.” The ROC curve always goes through the points of (0, 0) and (1, 1), indicating (sensitivity = 0, specificity = 1) and (sensitivity = 1, specificity = 0), respectively. In the case of point (0, 0) , it always gets the negative samples right but it gets all positive samples wrong due to the highest possible threshold. The point of (1, 1) corresponds to the lowest possible thresholds, classifying all of the samples as positive. The different shapes of the ROC curve provide different area under the curve (AUC) values, which is generally used as an indicator of the quality (discriminative ability) of a classifier. An AUC value close to 1 indicates a strong discriminative ability, and an AUC value close to 0.5 indicates that the classifier has little discriminative power. Incorporating the above evaluation parameters, this study also employed the Matthews Correlation Coefficient (MCC) (33) and Cohen's Kappa coefficient (KAPPA) (34) to provide a more comprehensive evaluation of the model's predictive performance. The MCC takes into account all four confusion matrix entries (*T*_*P*_, *T*_*N*_, *F*_*P*_ and *F*_*N*_), providing a balanced measure of model quality even if the classes are of very different sizes. The value ranges from −1 to 1, where 1 represents perfect prediction, 0 indicates random prediction, and −1 signifies total disagreement. Meanwhile, KAPPA was calculated to assess the agreement between the predicted classifications and the actual ground truth labels. It compares the observed agreement with the expected agreement by random chance. A Kappa value of 1 indicates perfect agreement, while a value of 0 implies that any observed agreement is purely due to chance. The PLS-LDA and PLS-DA models were established and evaluation parameters were calculated using Matlab2014a (Math works, Natick, MA, USA).

Competitive adaptive reweighted sampling (CARS) was used to select key wavelengths after optimal pretreatment of absorbance spectra data to simplify the procedure (35). The method is based on a simple but effective “survival of the fittest” principle. Key variables are defined as those with large absolute coefficients in a multivariate linear discriminant model. The absolute values of the regression coefficients estimated for model are used as an index to evaluate the importance of individual variables. The CARS method sequentially selects N subsets of variables from N Monte Carlo sampling runs in an iterative and competitive manner according to the importance of each variable. In each sampling run, some samples are first randomly chosen in a fixed ratio to build a calibration model. Next, the exponentially decreasing function and adaptive reweighted sampling processes select the key variables based on the regression coefficients. Finally, the subset with the lowest RMSECV is chosen. Details of the CARS procedure are available at https://libpls.net/.

**Figure 2:**
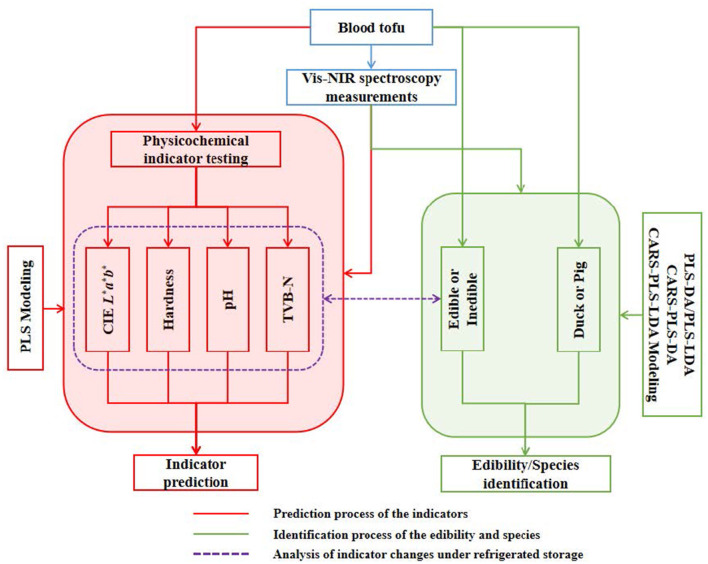
Schematic diagram of the data analysis pipeline.

Figure 2 illustrates the schematic diagram of the data analysis pipeline for this study.

## 3 Results

### 3.1 Changes and analysis of indicators under refrigerated storage

Table 1 presents data for duck blood tofu and pig blood tofu over a 15-day storage period. For both duck and pig blood tofu, TVB-N values increased. Compared to the early stage of storage, the rate of increase in TVB-N was faster in the later stage. This phenomenon might be attributed to a potentially faster rate of protein degradation or higher microbial load. The pH value for both species showed a gradual increase. The rise in pH was directly linked to the production of alkaline volatile compounds from protein degradation (as seen in the TVB-N increased) (36). However, compared with other physical and chemical indicators, the change in pH value was not particularly significant. Therefore, when evaluating the quality of blood tofu, the effect of pH value might not be particularly obvious. Pig blood deteriorated faster. Its TVB-N levels begun to rise significantly from day 3, and pH jumped on day 6. However, Duck blood remained relatively stable for the first 9 days, with significant deterioration only occurring after day 12. During storage, endogenous enzymes and microbial enzymes broke down proteins into peptides and amino acids, which were further deaminated or decarboxylated to form TVB-N such as ammonia and trimethylamine (37). The accumulation of these alkaline substances raised the pH value. The faster raised in TVB-N and pH in pig blood suggested higher enzymatic activity or a protein structure more susceptible to microbial utilization compared to duck blood. Both species experienced a decrease in lightness, a decrease in redness and an increase in yellowness. This suggested that hemoglobin in pig blood was less stable and more prone to oxidation. The formation of brown pigments (met-hemoglobin) and potential moisture lose or surface drying contributed to the darkening of the product. Blood tofu got its red color primarily from hemoglobin. During storage, hemoglobin oxidized from the bright red hemoglobin form to the darker brown met-hemoglobin form. This oxidation caused the significant drop in the a^*^ value. Meanwhile, increase in yellowness was often associated with the oxidation of lipids and the accumulation of yellowish breakdown products from proteins and heme groups. Duck blood naturally has a denser protein network structure compared to pig blood, which explains why duck blood tofu is inherently harder. However, pig blood tofu showed a larger relative percentage increase in hardness. The continued cross-linking or dehydration during storage exacerbates this firmness in both types. The increase in hardness was likely due to protein denaturation and aggregation continuing over time, even at refrigerated storage. As the protein network tightens and moisture is redistributed or lost, the texture becomes firmer. Over 15 days, both duck and pig blood tofu underwent protein degradation, oxidation of pigments, and textural hardening. Refrigerated storage slowed down but did not stop the deterioration processes. The changes of indexes of blood tofu under refrigerated storage were similar to those of meat. Duck blood tofu demonstrated superior overall storage stability compared to pig blood tofu. It maintained freshness, color, and texture for a longer period. Pig blood tofu suffered from early color fading and late-stage hardening. Therefore, the recommended shelf life was ≤ 6 days for pig blood and ≤ 9–10 days for duck blood in this experiment. Although these thresholds define the safe consumption period, data collected beyond these limits were not discarded during model development. Instead, they served as essential negative samples to train the model to recognize products that covered both within and beyond the recommended shelf life.

**Table 1:** Variation of physicochemical parameters of blood tofu samples under refrigerated storage.

Species	Physicochemical indexes	Storage time (days)					
		0	3	6	9	12	15
Duck	TVB-N (mg/100 g)	5.23 ± 1.94^a^	5.88 ± 0.95^a^	5.97 ± 2.56^a^	6.35 ± 1.04^a^	9.61 ± 0.89^b^	9.99 ± 1.74^b^
	pH	5.44 ± 0.09^a^	5.48 ± 0.07^a^	5.54 ± 0.13^ab^	5.54 ± 0.12^ab^	5.64 ± 0.16^bc^	5.70 ± 0.17^c^
	L^*^	37.07 ± 2.40^a^	36.14 ± 1.87^ab^	35.99 ± 2.18^ab^	35.86 ± 2.18^ab^	35.50 ± 0.77^b^	33.71 ± 0.80^c^
	a^*^	15.34 ± 2.38^a^	14.91 ± 2.37^a^	14.84 ± 1.79^a^	14.49 ± 1.56^a^	13.30 ± 0.62^ab^	12.46 ± 0.44^b^
	b^*^	10.74 ± 0.67^a^	11.11 ± 0.89^a^	11.41 ± 1.23^a^	11.56 ± 0.93^a^	11.73 ± 0.65^a^	12.17 ± 0.80^a^
	Hardness (N)	34.77 ± 2.59^a^	36.46 ± 3.80^a^	36.60 ± 2.39^a^	37.47 ± 2.91^a^	37.55 ± 1.97^a^	37.90 ± 3.58^a^
Pig	TVB-N (mg/100 g)	5.51 ± 1.34^a^	6.25 ± 1.04^ab^	7.47 ± 0.86^abc^	8.21 ± 1.66^bcd^	8.68 ± 0.95^cd^	10.27 ± 1.96^d^
	pH	5.58 ± 0.15^a^	5.61 ± 0.13^a^	5.75 ± 0.07^b^	5.78 ± 0.09^b^	5.79 ± 0.05^b^	5.82 ± 0.17^b^
	L^*^	30.63 ± 2.99^a^	29.97 ± 1.22^a^	29.82 ± 2.24^a^	29.70 ± 1.68^a^	29.60 ± 1.56^a^	28.89 ± 0.74^a^
	a^*^	15.89 ± 2.91^a^	15.26 ± 3.46^a^	13.35 ± 1.51^ab^	12.93 ± 2.06^ab^	12.92 ± 1.59^ab^	12.70 ± 1.08^b^
	b^*^	10.23 ± 1.25^a^	10.84 ± 0.73^a^	11.03 ± 0.72^a^	11.04 ± 0.53^a^	11.18 ± 0.75^a^	11.27 ± 1.25^a^
	Hardness (N)	16.95 ± 2.52^a^	18.36 ± 4.22^a^	19.63 ± 3.38^a^	20.88 ± 1.91^ab^	21.16 ± 1.32^ab^	22.54 ± 2.12^b^

Data are given as means ± standard deviation (SD). a, b, c, d, the same letter in the same row indicates homogeneous group established by the ANOVA (*P* < 0.05).

### 3.2 Analysis of Vis-NIR spectra

**Figure 3:**
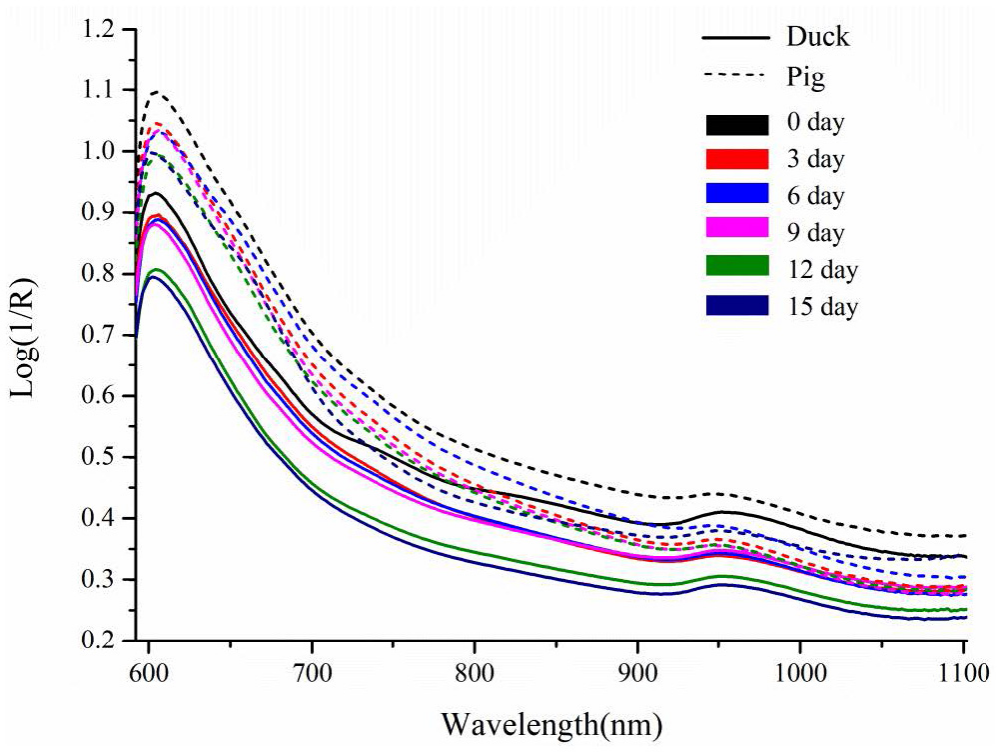
Typical Vis-NIR spectra for blood tofu samples.

Figure 3 shows average Vis-NIR reflectance spectra of duck blood tofu (solid lines) and pig blood tofu (dashed lines) stored under refrigerated conditions over a 15-day period (0, 3, 6, 9, 12, and 15 days). A progressive decrease in $Log\left(\frac{1}{R}\right)$ values was observed across the full spectral range, especially in the visible region, as storage duration increased. This suggested the continuous degradation of chromophores, even under relatively low-temperature preservation. Pig blood tofu maintained consistently higher $Log\left(\frac{1}{R}\right)$ levels than duck blood tofu across all time points, suggesting greater initial chromophore density or altered degradation rates. Significantly, the spectral gap between species widened markedly after day 6, underscoring distinct stability profiles under refrigeration.

As illustrated in the Figure 3, pig blood tofu exhibited significantly stronger absorption in the visible region, indicative of a potentially higher concentration of heme-bound iron. Notably, compared to duck blood tofu, pig blood tofu displayed a more pronounced decline in absorbance within the 600–700 nm range, suggesting a greater susceptibility to browning. The 700–900 nm range, corresponding to the short-wave NIR region, is sensitive to vibrational overtones and combinations of C-H, N-H, and O-H bond (38). Variations in these functional groups reflect alterations in protein conformation and lipid-protein interactions. The steeper spectral slope observed in pig samples might imply an accelerated rate of lipid oxidation. Furthermore, the 900–1100 nm region characterizes the second overtone of O-H stretching vibrations, providing insights into water binding states and gel matrix properties (39). Duck blood tofu demonstrated a more stable and gradual spectral profile, which might be attributed to its denser microstructural network. These spectroscopic findings were consistent with the aforementioned physicochemical analysis results. Crucially, the superior stability of duck blood tofu in terms of both color and texture could inadvertently incentivize unethical practices by manufacturers, such as product adulteration or the unauthorized extension of shelf-life.

### 3.3 Prediction of parameters by Vis-NIR spectroscopy

The results of Table 2 describes the performance of PLS regression models developed to predict the physicochemical indicators of duck blood tofu and pig blood tofu using spectral data. To achieve optimal prediction performance, distinct spectral pre-processing techniques were applied for different blood tofu species and quality indices. The 1^st^ was the most frequently employed method, primarily used to eliminate baseline drift and light scattering effects. The 2^nd^ was used to enhance spectral resolution and separate overlapping peaks. SNV transformation was mainly utilized to mitigate scattering effects and particle size variations. MSC, similar to SNV, was applied to correct for light scattering. The number of LVs selected for the models ranged from 5 to 7, consistently remaining below 10. This indicated that the models extracted the 5–7 most significant components from the spectral data to explain the variations in physicochemical indices. Furthermore, the use of a relatively low number of LVs suggested that the models possessed stable predictive capabilities. Based on a comprehensive analysis of the various evaluation parameters, the models for the color parameters were suitable for quantitative analysis (40, 41), especially for L^*^ and a^*^. For both duck and pig blood tofu, the *R*_c_ for color parameters exceeded 0.90, and the *R*_p_ were approached or exceeded 0.90. RPD values were approached or exceeded 2.0, and RER values were greater than 6.0. In comparison to color parameters, the modeling results for hardness, TVB-N, and pH were relatively inferior, suggesting that these three indices were more challenging to capture precisely using Vis-NIR spectroscopy. However, the performance of TVBN remained within an acceptable range, which was classic indicators for evaluating the freshness of meat by-products. Additionally, the Table 2 revealed that duck and pig blood tofu required different spectral pre-processing methods for model establishment. This discrepancy was likely related to differences in their intrinsic protein structures, densities, and light scattering properties (as indicated in Table 1, where the hardness of duck blood was significantly higher than that of pig blood). The aforementioned results indicate that Vis-NIR spectroscopy technology holds considerable potential for the quality detection of blood tofu's color parameters.

**Table 2:** Modeling results for quality parameters of blood tofu samples using PLS models derived from Vis-NIR data.

Species	Physicochemical indexes	Modeling results									
		Pretreatment	LVs	*R*_c_	RMSEC	RPD_cv_	RER_cv_	*R*_p_	RMSEP	RPD	RER
Duck	TVB-N (mg/100 g)	1^st^	5	0.9021	0.856	1.22	4.27	0.8692	1.02	1.93	5.49
	pH	1^st^	6	0.8366	0.0653	0.73	3.33	0.7418	0.0829	1.38	5.04
	L^*^	1^st^	5	0.9463	0.695	1.99	4.01	0.8660	0.794	1.91	6.52
	a^*^	SNV+1^st^	5	0.9675	0.495	2.59	11.04	0.8989	0.517	2.34	9.42
	b^*^	1^st^	7	0.9502	0.295	1.29	6.06	0.8495	0.384	1.80	7.58
	Hardness (N)	1^st^	7	0.9054	1.13	1.49	5.59	0.8337	1.18	1.80	5.44
Pig	TVB-N (mg/100 g)	1^st^	5	0.8545	0.774	0.95	4.46	0.8247	1.04	1.66	5.38
	pH	2^nd^	6	0.9161	0.0503	1.15	5.64	0.8396	0.0676	1.79	5.32
	L^*^	1^st^	5	0.9708	0.509	2.90	12.97	0.8338	0.567	2.46	12.29
	a^*^	SNV	6	0.9664	0.641	3.23	14.42	0.9074	0.676	2.09	8.77
	b^*^	1^st^	7	0.9058	0.313	1.10	5.30	0.8578	0.359	1.78	7.94
	Hardness (N)	MSC + 2^nd^	7	0.9066	1.22	0.92	3.77	0.8440	1.92	1.30	3.93

1^st^, first derivative; 2^nd^, second derivative; SNV, standard normal variate; MSC, multiplicative scatter correction; CV, cross-validation, *R* correlation coefficient; RMSEC, root mean square error of calibration; RMSEP, root mean square error of prediction; RPD, ratio of prediction to deviation; RER, range error ratio.

### 3.4 Blood tofu species identification by Vis-NIR spectroscopy

**Figure 4:**
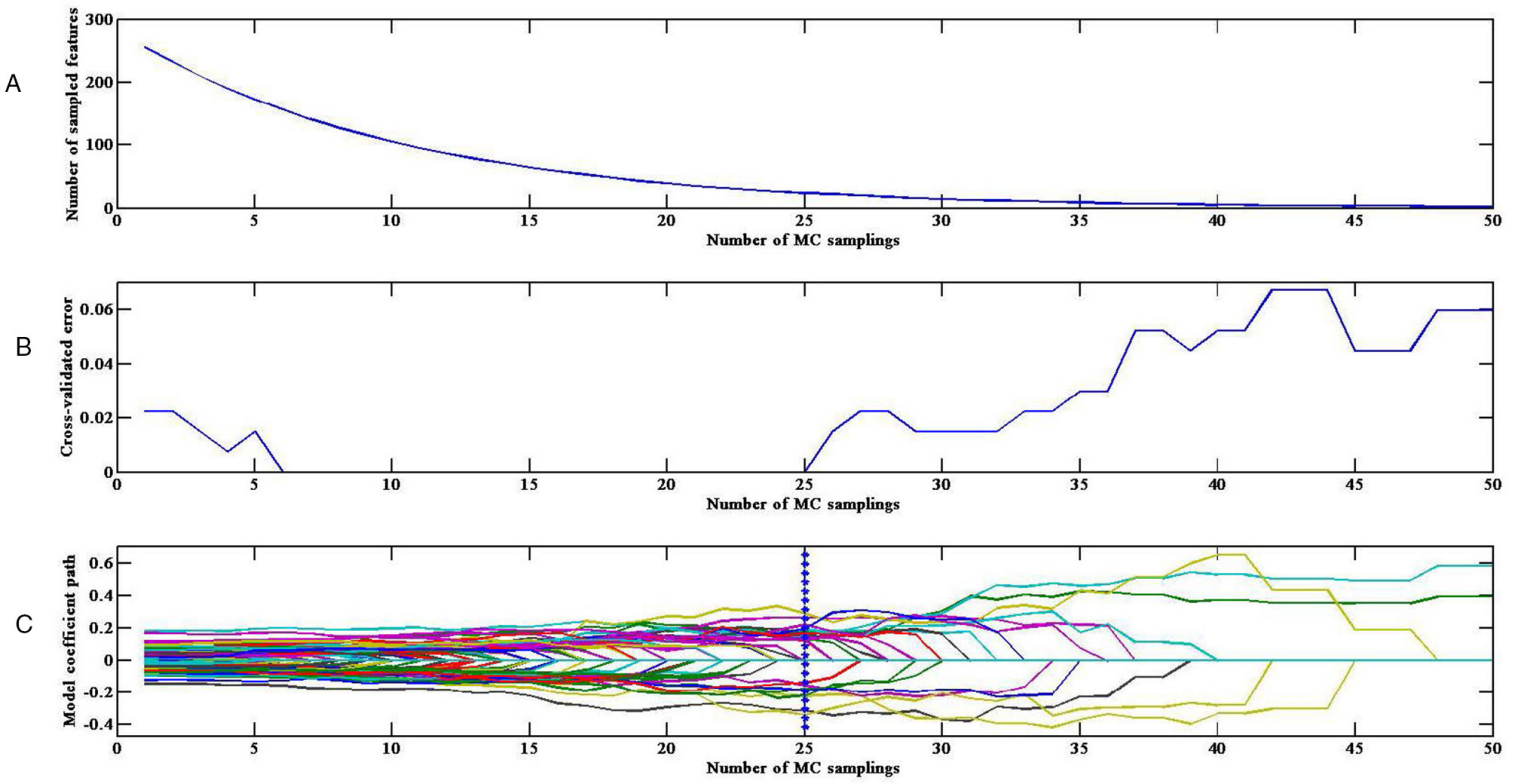
Variables selection by CARS for absorption spectra after first-derivative pretreatment. **(A)** The changes of the number of selected wavelengths, **(B)** the changes of RMSECV, **(C)** the regression coefficients of each wavelength during the calculations of the CARS algorithm.

The samples of duck blood tofu and pig blood tofu were individually divided into calibration and validation sets in a ratio of 3–1 using the Kennard-Stone (KS) algorithm. Subsequently, the respective calibration sets and validation sets from both types of blood tofu were pooled to generate a combined calibration set and a combined validation set. A total of 134 samples (67 duck blood tofu samples and 67 pig blood tofu samples) were assigned to the calibration set, while the validation set contained 46 (23 duck blood tofu samples and 23 pig blood tofu samples). Firstly, PLS-LDA and PLS-DA models were developed based on using full-spectrum data for the species identification between duck blood tofu and pig blood tofu. And then, CARS was used to select key wavelengths to develop species identification model. Taking the development of the CARS-PLS-LDA model as a representative example, Figure 4 illustrates the iterative variable selection process of the CARS algorithm. Figure 4A shows the decreasing number of wavelengths as Monte Carlo samplings increase, reflecting progressive elimination of redundant or noisy variables; the Figure 4B plots the cross-validated error, which typically decreases initially and then rises after over-thinning—indicating an optimal subset near the error minimum (around 25 Monte Carlo samplings, marked by the vertical dashed line); the Figure 4C displays the evolution of regression coefficients for each wavelength across iterations, where stable, non-zero coefficients indicate informative wavelengths retained for the final PLS-LDA model.

**Figure 5:**
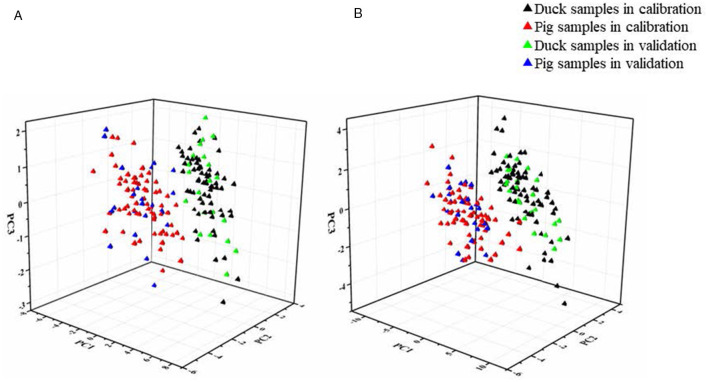
Score distribution of the first three principal components. **(A)** CARS-PLS-LDA. **(B)** CARS-PLS-DA.

Table 3 presents the performance of both full-spectrum and CARS models following 2^nd^ pre-processing. Compared to the full-spectrum models, the CARS-optimized models exhibited superior classification accuracy in both calibration and validation sets, with specificity, sensitivity, AUC, MCC, and Kappa coefficients approaching or reaching 1.00. This improvement is attributed to the retention of key chemometric information relevant to species differentiation. Table 4 shows details the specific wavelengths selected by the CARS algorithm. The CARS-PLS-LDA and CARS-PLS-DA utilized only 24 and 39 characteristic wavelengths, respectively (approximately 9 and 15% of the full spectrum). This clear demarcation indicated that the selected wavelengths captured intrinsic biochemical differences between the two species' blood matrices—particularly in hemoglobin structure (Soret band region: 608–674 nm, sensitive to heme iron oxidation state and globin conformation) (42), protein–water interactions (850–914 nm, reflecting hydrogen-bonding networks in coagulated matrix) (43), and lipid composition (978–1086 nm, associated with acyl chain C–H overtones) (44). Given that blood tofu is produced via thermal coagulation of whole blood, these spectral distinctions likely originate from species-dependent variations in hemoglobin isoforms, plasma protein profiles, and endogenous lipid content—factors known to influence gel strength, color stability, and textural properties in coagulated blood products. Furthermore, under the identical variable selection strategy, CARS-PLS-LDA demonstrated slightly superior performance compared to CARS-PLS-DA. Figure 5 illustrates the 3D scores plots of the first three principal components (PCs) for the CARS-PLS-LDA and CARS-PLS-DA models. In both plots, PC1 serves as the primary axis for species discrimination, with duck blood samples predominantly clustering in the positive region and pig blood samples in the negative region. Conversely, PC2 and PC3 primarily reflect intra-class variations, potentially associated with quality deterioration during refrigerated storage. While the CARS-PLS-DA plot shows a general separation trend, there is a slight overlap between pig (red) and duck (green) samples near the PC1 decision boundary. In contrast, the CARS-PLS-LDA plot exhibits a distinct separation boundary, with samples displaying a more compact distribution along PC2 and PC3. This indicates that the CARS-PLS-LDA more effectively maximized inter-class variance while minimizing intra-class variance, demonstrating superior efficiency in handling non-discriminative spectral variations compared to the CARS-PLS-DA. The CARS-PLS-LDA model utilized only 3 LVs, and the 3D score plot demonstrates that the validation samples are distributed closely with the calibration samples and are clearly separated. The robustness of the model across both calibration and validation sets underscores its potential as a rapid, non-destructive tool for verifying species origin in commercial blood-based meat by-products or traditional blood sausages, thereby supporting traceability and combating economic adulteration in meat processing chains.

**Table 3:** Blood tofu species identification models based on full-spectrum 2^nd^ pre-treatment.

Model	Number of wavelengths	LVs	Sensitivity (%)	Specificity (%)	AUC	*C*_cal_(%)	MCC	KAPPA	*C*_val_ (%)	MCC	KAPPA
PLS-LDA	256	4	100.00	97.51	1.00	98.51	0.97	0.98	100.00	1.00	1.00
CARS-PLS-LDA	24	3	100.00	100.00	1.00	100.00	1.00	1.00	100.00	1.00	1.00
PLS-DA	256	9	98.51	98.51	0.99	98.51	0.97	0.97	95.65	0.91	0.91
CARS-PLS-DA	39	5	100.00	100.00	1.00	100.00	1.00	1.00	97.83	0.96	0.96

**Table 4:** Selected wavelengths by CARS for blood tofu species identification models.

Model	Wavelength (nm)
CARS-PLS-LDA	608, 612, 614, 632, 664, 670, 674, 696, 798, 850, 868, 874, 880, 904, 912, 914, 936, 938, 958, 960, 978, 1012, 1062, 1086
CARS-PLS-DA	608, 612, 614, 620, 632, 640, 670, 674, 724,796, 798, 804, 824, 836, 870,916, 928, 936, 938, 946,950, 958, 960, 968,970, 972, 976, 978, 980, 988, 992, 1000, 1006, 1010, 1012, 1028, 1046, 1052, 1062

### 3.5 Development and analysis of discriminant models for edible blood tofu

Based on the results presented in Table 1 and the subsequent data analysis, duck blood tofu samples stored for more than 9 days and pig blood tofu samples stored for more than 6 days were defined as not recommended for consumption. Accordingly, for duck blood tofu, 60 samples stored under refrigeration for ≤ 9 days were classified as edible blood tofu, while 30 samples stored for ≥12 days were classified as inedible. Similarly, for pig blood tofu, 45 samples stored for ≤ 6 days were categorized as edible, whereas the remaining 45 samples stored for ≥9 days were deemed inedible. Then, data sets within each of the species data sets were created, as calibration and validation sets in a ratio of 3–1, respectively, using the KS method. Consequently, both PLS-LDA and PLS-DA models also were established to distinguish between samples recommended for consumption and those not recommended, with the results summarized in Table 5.

**Table 5:** Models based on full-spectrum 2^nd^ pre-treatment for distinguishing edible and inedible blood tofu.

Species	Method	Number of wavelengths	LVs	Sensitivity (%)	Specificity (%)	AUC	*C*_cal_(%)	MCC	KAPPA	*C*_val_ (%)	MCC	KAPPA
Duck	PLS-LDA	256	3	93.18	100.00	0.99	95.52	0.94	0.94	91.30	0.68	0.68
	CARS-PLS-LDA	12	2	100.00	100.00	1.00	100.00	1.00	1.00	100.00	1.00	1.00
	PLS-DA	256	4	97.67	100.00	0.96	97.67	0.97	0.97	95.83	0.90	0.89
	CARS-PLS-DA	71	3	100.00	100.00	1.00	100.00	1.00	1.00	95.65	0.90	0.90
Pig	PLS-LDA	256	3	96.97	97.06	0.99	97.01	0.85	0.85	100.00	1.00	1.00
	CARS-PLS-LDA	13	2	100.00	100.00	1.00	100.00	1.00	1.00	100.00	1.00	1.00
	PLS-DA	256	3	96.97	97.06	0.95	97.01	0.94	0.95	100	1.00	1.00
	CARS-PLS-DA	53	4	100.00	100.00	1.00	100.00	1.00	1.00	95.65	0.92	0.90

AUC, area under the curve; *C*_cal_, classification accuracy in calibration set; *C*_val_, classification accuracy in validation set; MCC, Matthews Correlation Coefficient; KAPPA, Cohen's Kappa coefficient.

First, the discriminant models established based on the full-spectrum 2^nd^ pre-treatment exhibited good discrimination performance for both duck blood tofu and pig blood tofu samples. The low LVs in the full-spectrum models indicated a relatively parsimonious model structure that was less prone to overfitting. Meanwhile, the sensitivity and specificity values were all close to or equal to 100%, demonstrating that the models could perfectly identify “edible” (sensitivity) and “inedible” (specificity) samples within the calibration set, with virtually no false positives or false negatives. Furthermore, the AUC values approached or reached 1.00, indicating high discriminative power. Meanwhile, the model exhibited high MCC and Kappa coefficients (≥0.85), indicating a balanced discriminative ability for both positive and negative classes, as well as a high consistency between the predicted results and the actual labels. However, as shown in Table 5, the performance on the calibration set for pig blood tofu was slightly inferior to that of the validation set. Statistical analysis suggested that the calibration set contained certain edge samples. Spectral noise and irrelevant information also contributed to the misclassification of these samples. Meanwhile, the results demonstrated that the model achieved comprehensive and robust learning on the calibration set, thereby maintaining strong predictive capability when applied to the validation set.

Building upon the full-spectrum models, the models established using wavelengths selected via the CARS method achieved superior performance with fewer variables. Compared with the full-spectrum model, the evaluation parameters of the model were all improved after screening the wavelength variables. This demonstrated that screening for characteristic wavelengths effectively removed redundant information, thereby simplifying the model and enhancing computational efficiency. Notably, some literatures revealed that the majority of these selected variables (shown in Supplementary Material Table) were associated with specific absorption peaks or characteristic vibrations of key chemical constituents (e.g., hemoglobin, proteins, and moisture) (45–48), providing a solid theoretical basis for the variable selection.

Through comparative analysis, it was observed that PLS-DA slightly outperformed PLS-LDA in full-spectrum modeling, demonstrating superior adaptability to high-dimensional and redundant spectral data. Comparative analysis revealed that PLS-DA slightly outperformed PLS-LDA in full-spectrum modeling, demonstrating superior adaptability to high-dimensional and redundant spectral data. However, after variable selection via CARS, LDA effectively utilized its linear decision boundaries to construct clear class separations, thereby ensuring higher discrimination accuracy.

## 4 Discussion

This study successfully established Vis-NIR spectroscopy models to identify the species (duck vs. pig) and edibility of blood tofu. As shown in Table 5, full-spectrum PLS-DA slightly outperformed or equaled PLS-LDA to identify edible and inedible samples before variable selection. This is because PLS-DA maximizes covariance between spectra and class labels, offering better tolerance for non-linear data. In contrast, PLS-LDA relies on a linear separability assumption by maximizing inter-class variance. However, the CARS significantly improved model performance, with CARS-PLS-LDA emerging as the optimal model in this study. By eliminating irrelevant variables and background noise, CARS retained fewer key wavelengths. This purification made the data distribution more suitable for LDA's linear separation. Figure 5 visually confirms this: the CARS-PLS-LDA scores plot shows complete separation along PC1 with tight intra-class clustering, indicating effective suppression of intra-class variation. Conversely, the CARS-PLS-DA plot exhibits slight overlap at the PC1 boundary, suggesting that without sufficient feature purification, PLS-DA yields less robust classification boundaries. Thus, CARS-PLS-LDA is the better qualitative model due to its parsimonious structure and clear class discrimination in this study.

Regarding the quantitative prediction of quality indicators during refrigerated storage (Table 2), except for color parameters, the predictive accuracy for TVB-N, pH, and hardness was suboptimal. CARS-based variable selection did not yield better performance than the full-spectrum models, its results were reported in Supplementary Material. This outcome can be attributed to two main factors: firstly, Vis-NIR spectroscopy is an indirect measurement technique that can't directly detect the molecular vibrations of indicators, such as TVB-N. Instead, it captures spectral variations resulting from underlying biochemical reactions like protein degradation and moisture loss (49). These indirect correlations are typically distributed across broad spectral regions as weak, overlapping signals. Although full-spectrum models contain noise, they preserve all potentially relevant weak information. PLS excels at handling high-dimensional collinear data by capturing this cumulative spectral effect through latent variables. Conversely, CARS may inadvertently eliminate wavelengths that make small but meaningful contributions to regression, leading to a loss of critical information and reduced predictive power. Secondly, the regression of continuous variables is sensitive to sample coverage. Given the limited sample size in this study, the full-spectrum model leverages a larger number of data points to construct the regression equation, acting as a form of regularization to enhance robustness (50).

## 5 Conclusion

Based on the comprehensive analysis of physicochemical, sensory, and spectral data presented in this study, the following conclusions can be drawn regarding the application of portable Vis-NIR spectroscopy for blood tofu quality assessment. The technology demonstrated promising feasibility as a rapid, non-destructive tool for monitoring key quality parameters during refrigerated storage, with particularly robust performance for color prediction. Critically, PLS-LDA and PLS-DA discriminant models, especially those utilizing CARS-selected wavelengths, achieved high classification accuracy in distinguishing edible from inedible samples, suggesting the potential of this approach for shelf-life evaluation under refrigerated storage. Furthermore, the method successfully discriminated between duck and pig blood tofu origins with excellent accuracy, highlighting its utility for species identification and its potential contribution to preventing mislabelling in meat by-products supply chains. Although this study has yielded promising results, several challenges must be addressed to further promote the extensive application of this technology in the food sector. Future research should prioritize expanding the sample size and incorporating samples from diverse geographical origins and processing methods to enhance model robustness. These findings collectively establish a solid foundation for the further development of Vis-NIR spectroscopy as an effective quality control and traceability tool for blood-based meat by-products analogs.

## Data Availability

The original contributions presented in the study are included in the article/Supplementary material, further inquiries can be directed to the corresponding author.
