# Supplementary material for: Non-destructive quality assessment and species identification of blood tofu using portable visible/near-infrared spectroscopy
Source: Front Nutr. 2026 Jul 3;13:1877866. doi: 10.3389/fnut.2026.1877866 (PMC13375554; doi:10.3389/fnut.2026.1877866)
Supplement: Supplementary file 1 [file Table_1.docx]

| Species | Physicochemical indexes |  |  | Modeling results | | | | | |  |  |
| --- | --- | --- | --- | --- | --- | --- | --- | --- | --- | --- | --- |
|  |  | Pretreatment | LVs | *R*_c_ | RMSEC | RPD_cv_ | RER_cv_ | *R*_p_ | RMSEP | RPD | RER |
| Duck | TVB-N (mg/100 g) | 1^st^ | 5 | 0.9236 | 0.958 | 1.14 | 6.29 | 0.8844 | 1.185 | 1.35 | 5.45 |
|  | pH | 1^st^ | 6 | 0.7225 | 0.114 | 1.46 | 5.70 | 0.6084 | 0.175 | 0.92 | 3.96 |
|  | *L^*^* | 1^st^ | 5 | 0.9477 | 0.659 | 1.65 | 6.35 | 0.8890 | 0.899 | 1.07 | 4.12 |
|  | *a^*^* | SNV+1^st^ | 5 | 0.9279 | 0.718 | 1.76 | 6.76 | 0.9077 | 1.003 | 1.42 | 4.51 |
|  | *b^*^* | 1^st^ | 7 | 0.9735 | 0.237 | 1.99 | 8.50 | 0.8274 | 0.467 | 1.45 | 6.80 |
|  | Hardness (N) | 1^st^ | 7 | 0.9592 | 0.828 | 1.91 | 8.30 | 0.8282 | 1.65 | 1.62 | 6.08 |
| Pig | TVB-N (mg/100 g) | 1^st^ | 5 | 0.9235 | 0.678 | 2.16 | 9.59 | 0.7409 | 1.103 | 1.51 | 6.35 |
|  | pH | 2^nd^ | 6 | 0.8466 | 0.0784 | 1.66 | 9.91 | 0.7540 | 0.0869 | 1.16 | 4.37 |
|  | *L^*^* | 1^st^ | 5 | 0.9071 | 0.773 | 1.70 | 6.88 | 0.8756 | 0.781 | 1.86 | 6.69 |
|  | *a^*^* | SNV | 6 | 0.8853 | 1.23 | 1.68 | 8.16 | 0.6275 | 0.90 | 1.52 | 6.52 |
|  | *b^*^* | 1^st^ | 7 | 0.9590 | 0.262 | 2.18 | 10.98 | 0.7354 | 0.513 | 1.19 | 4.15 |
|  | Hardness (N) | MSC+2^nd^ | 7 | 0.9354 | 1.18 | 1.73 | 8.14 | 0.8506 | 2.300 | 1.48 | 5.38 |

Supplementary TABLE 1 PLS modeling results for quality parameters of blood tofu using Vis-NIR data with CARS-selected wavelengths

Supplementary TABLE 2 Selected wavelength by CARS for distinguishing edible and inedible blood tofu

| Species | Method | Selected wavelength (nm) |
| --- | --- | --- |
| Duck | CARS-PLS-LDA | 610, 690, 772, 774, 796, 832, 846, 954, 966, 988, 1034, 1042 |
|  | CARS-PLS-DA | 602,606,608,610,622,624,628,636,642,644,650,652,654,656,660,672,674,682,686,690,696,702,706,708,714,716,722,724,726,758,762,766,774,788,792,796,810,836,888,904,912,920,928,946,952,960,966,970,972,984,992,1000,1002,1010,1018,1020,1022,1024,1030,1032,1034,1038,1044,1052,1054,1056,1066,1080,1082,1084,1088 |
| Pig | CARS-PLS-LDA | 636, 638, 642, 660, 700, 750, 764, 814, 870, 886, 894, 966, 1066 |
|  | CARS-PLS-DA | 606, 610, 622, 624, 628, 636, 646, 658, 660, 662, 674, 680,684,688,694,704,708,726,750, 764,778,782,806,924,932,934,940,944,946,952,962,966,984,990,994,996,1000,1004,1006,1012,1020,1024, 1038,1042,1044, 1050,1052,1054, 1060,1062,1074,1088, 1090 |
