# Supplementary material for: Non-destructive quality assessment and species identification of blood tofu using portable visible/near-infrared spectroscopy
Source: Front Nutr. 2026 Jul 3;13:1877866. doi: 10.3389/fnut.2026.1877866 (PMC13375554; doi:10.3389/fnut.2026.1877866)
Supplement: Supplementary file 2 [file Image_1.pdf]

Supplementary Material

1.1 Supplementary Figures

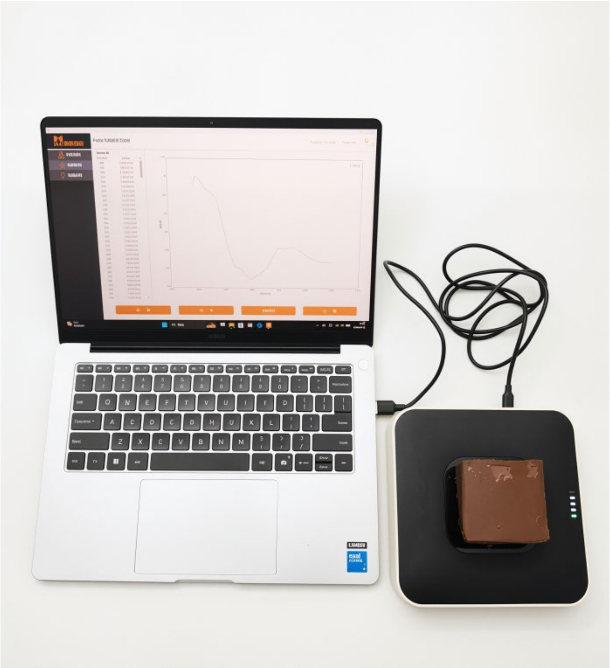

FIGURE 1 Vis-NIR spectroscopy measurement of blood tofu

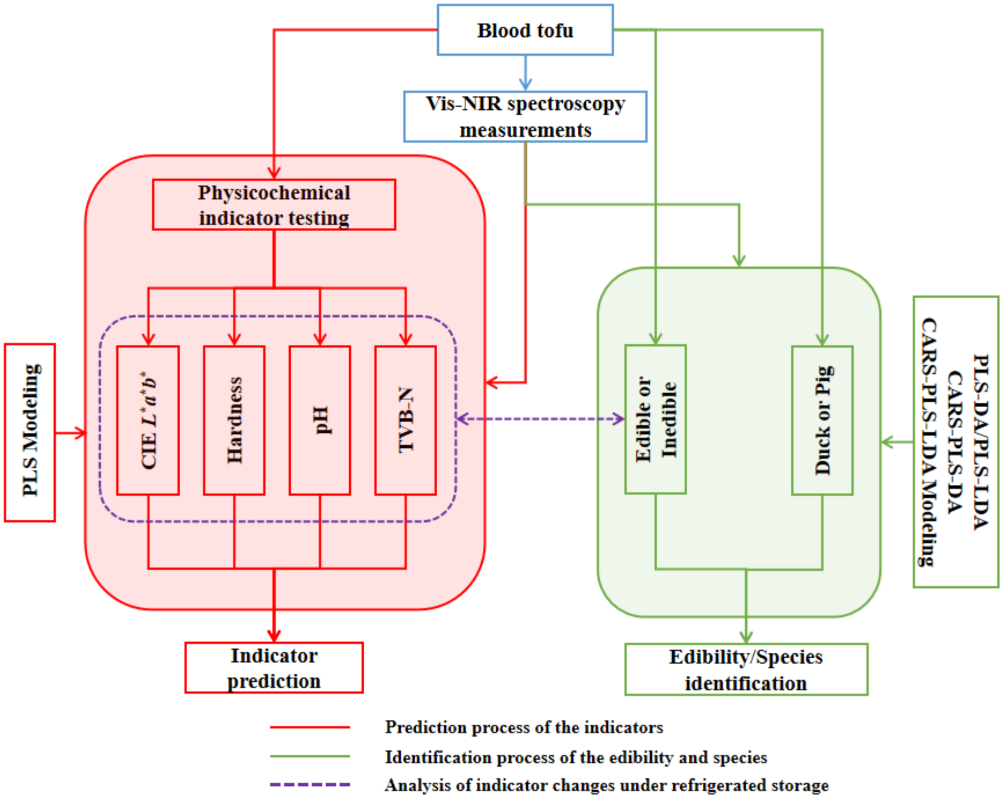

FIGURE 2 Schematic diagram of the data analysis pipeline

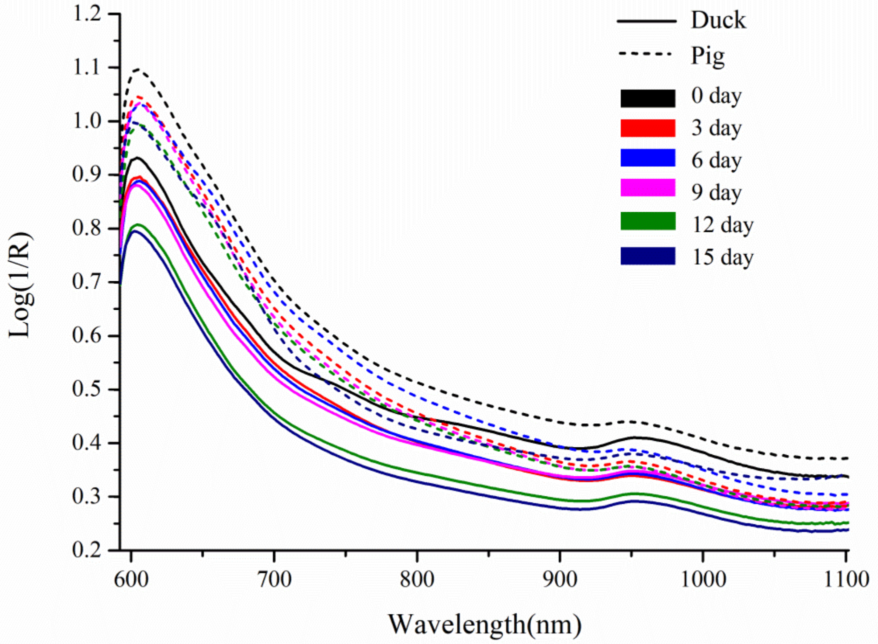

FIGURE 3 Typical Vis-NIR spectra for blood tofu samples

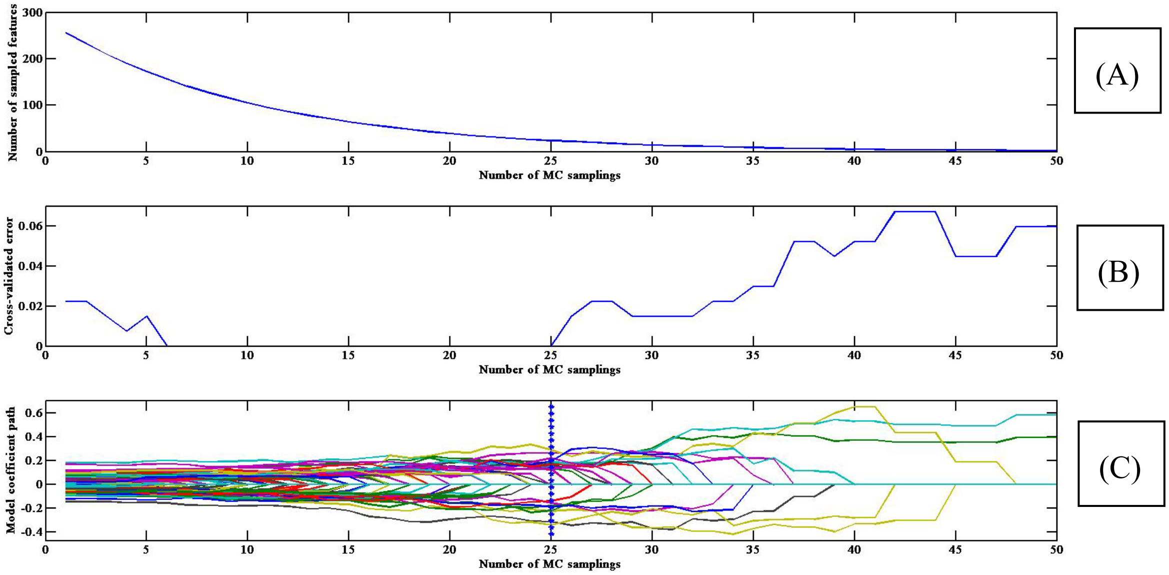

FIGURE 4 Variables selection by CARS for absorption spectra after first-derivative pretreatment.

(A) The changes of the number of selected wavelengths, (B) The changes of RMSECV, (C) The regression coefficients of each wavelength during the calculations of the CARS algorithm.

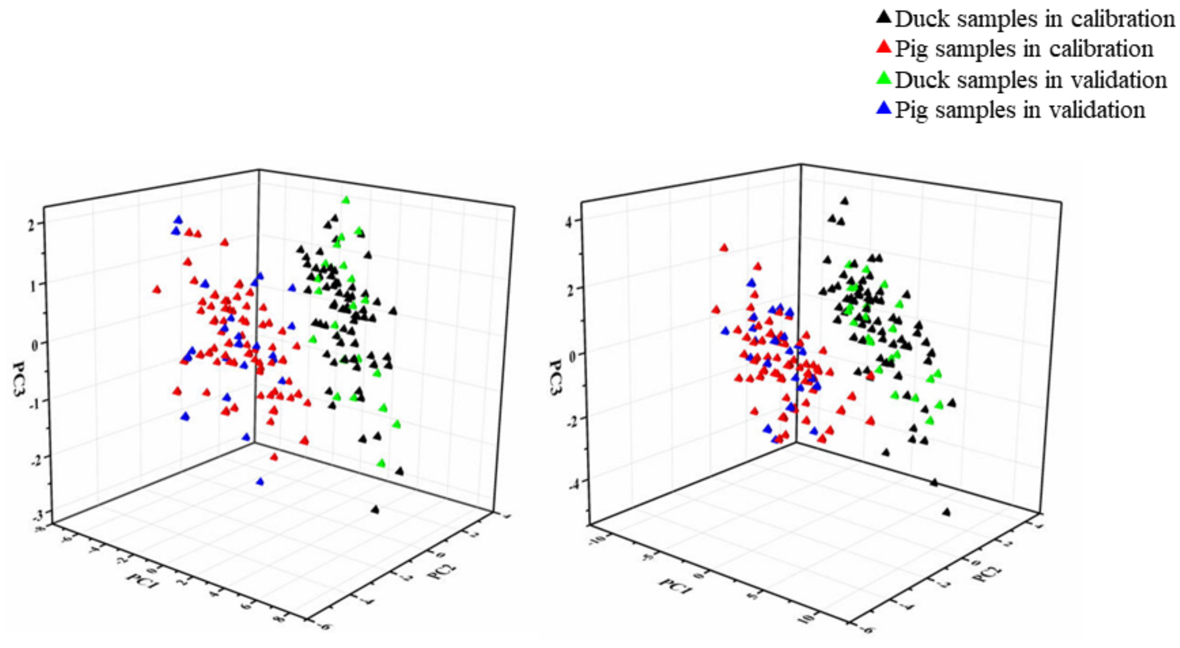

(A) (B)

FIGURE 5 Score distribution of the first three principal components

(A)CARS-PLS-LDA (B) CARS-PLS-DA
